# The Toxicity of Wiped Dust and Airborne Microbes in Individual Classrooms Increase the Risk of Teachers’ Work-Related Symptoms: A Cross-Sectional Study

**DOI:** 10.3390/pathogens10111360

**Published:** 2021-10-21

**Authors:** Janne Salin, Pasi Ohtonen, Maria A. Andersson, Hannu Syrjälä

**Affiliations:** 1 The Department of Infection Control, Oulu University Hospital, FI-90029 Oulu, Finland; hannu.syrjala@ppshp.fi; 2 Division of Operative Care, Oulu University Hospital, FI-90220 Oulu, Finland; pasi.ohtonen@oulu.fi; 3 Research Unit of Surgery, Anesthesia and Intensive Care, University of Oulu, FI-90014 Oulu, Finland; 4 Department of Microbiology, Faculty of Agriculture and Forestry, University of Helsinki, FI-00014 Helsinki, Finland; maria.a.andersson@helsinki.fi; 5 Department of Civil Engineering, Aalto University, FI-00076 Aalto, Finland

**Keywords:** building-related symptoms, indoor toxicity, boar sperm motility inhibition assay, mitochondrial toxicity, mitochondrial dysfunction

## Abstract

Background: The causes and pathophysiological mechanisms of building-related symptoms (BRS) remain open. Objective: We aimed to investigate the association between teachers’ individual work-related symptoms and intrinsic in vitro toxicity in classrooms. This is a further analysis of a previously published dataset. Methods: Teachers from 15 Finnish schools in Helsinki responded to the symptom survey. The boar sperm motility inhibition assay, a sensitive indicator of mitochondrial dysfunction, was used to measure the toxicity of wiped dust and cultured microbial fallout samples collected from the teachers’ classrooms. Results: 231 teachers whose classroom toxicity data had been collected responded to the questionnaire. Logistic regression analysis adjusted for age, gender, smoking, and atopy showed that classroom dust intrinsic toxicity was statistically significantly associated with the following 12 symptoms reported by teachers (adjusted ORs in parentheses): nose stuffiness (4.1), runny nose (6.9), hoarseness (6.4), globus sensation (9.0), throat mucus (7.6), throat itching (4.4), shortness of breath (12.2), dry cough (4.7), wet eyes (12.7), hypersensitivity to sound (7.9), difficulty falling asleep (7.6), and increased need for sleep (7.7). Toxicity of cultured microbes was found to be associated with nine symptoms (adjusted ORs in parentheses): headache (2.3), nose stuffiness (2.2), nose dryness (2.2), mouth dryness (2.8), hoarseness (2.2), sore throat (2.8), throat mucus (2.3), eye discharge (10.2), and increased need for sleep (3.5). Conclusions: The toxicity of classroom dust and airborne microbes in boar sperm motility inhibition assay significantly increased teachers’ risk of work-related respiratory and ocular symptoms. Potential pathophysiological mechanisms of BRS are discussed.

## 1. Introduction

Building-related symptoms (BRS) have been reported worldwide for decades [1]. Classic BRS include eye, nose, respiratory, and skin symptoms, as well as headache, fatigue, dizziness, inability to concentrate, nausea, fever, and chills [2,3]. In addition, exposure to moisture damage in buildings and emissions from indoor mold growth have been shown to be associated with the development and exacerbation of asthma, allergic rhinitis, and bronchitis [4,5,6]. However, the pathophysiological mechanisms and causal relationships of symptoms remain unclear.

In moisture-damaged buildings, a variety of toxic fungi and bacteria are known to grow in the structures, ventilation systems and on indoor surfaces [7,8,9,10,11,12]. Airborne dust, spores, and hyphae fragments can act as carriers of microbial toxins in indoor air [13,14,15]. Recent studies show that toxins can also be very easily aerosolized [16,17], particularly even without airflow, due to highly toxic guttation droplets secreted by microbes actively growing on building materials [10,11,18,19,20]. Settled dust collected from moisture-damaged buildings has been found to contain microbial toxins [21,22,23,24,25,26,27,28].

In toxicity measurements, various dust sampling and processing methods and different in vitro models have demonstrated contradictory results [16,29,30,31,32]. The inflammatory potential of the deposited dust in human lung epithelial cell A549 assay was statistically significantly associated with employees’ symptoms in schools [33] and offices [29]. Although the immunotoxicity of indoor samples in the mouse RAW264.7 macrophage assay has been described to reduce after the renovation of moisture-damaged schools [31,34], it was not possible to distinguish moisture-damaged schools from control schools with this method [32,35]. The same holds true for hemolytic activity tested on human erythrocytes or oxidative capacity by plasmid scission assay [36].

The classic boar sperm motility inhibition (BSMI) assay has been found to be a sensitive method for detecting bacterial and mycotoxins in moisture-damaged buildings [9,11,16,37,38,39,40,41,42,43,44,45,46]. The method has also shown to be sensitive to man-made xenobiotic mitochondriotoxic chemicals known to occur in indoor dusts [47,48]. In a recent study, a clear temporal association was found between heavy occupational exposure to sperm-toxic dust during renovation of a water-damaged building and a cluster of 21 new occupational asthma cases [49].

We previously showed that both the total number of literature-known BRS [50] and the most common other work-related symptoms [51] were associated with intrinsic in vitro toxicity of settled dust and cultivated airborne microbes from teachers’ classrooms. The aim of this sub-analysis of the same dataset was to investigate whether there is an association between teachers’ individual symptoms and the toxicity of dust and airborne microbes

## 2. Results

Two hundred and thirty-one teachers met the admission criteria, and had completed a questionnaire, and had at least one classroom toxicity result (200 respondents had microbial toxicity results and 169 respondents had dust toxicity results) [50,51]. The median age of the respondents was 43 years, 81.8% were women, 9.5% were current smokers, and 10.4% were atopic. Their median working time at their primary workplace was 22 h per week.

Table 1 shows the prevalence and association with the workplace of the literature-known BRS and the most common other symptoms (prevalence over 10%), of which at least 50% were perceived to be work-related. There was a total of 41 such symptoms, including 7 general, 17 respiratory, 3 dermal, 6 ocular, 2 hearing, 3 sleeping, and 3 mental symptoms. The highest work-relatedness (at least 70% of the symptom worsening in the workplace) was reported for eight symptoms: three general symptoms (fatigue, generalized feeling of sickness, indefinite feeling of thermoregulation failure), four respiratory symptoms (hoarseness, dry cough, throat mucus, throat itching), and one mental symptom (mental irritability).

**Figure 1:**
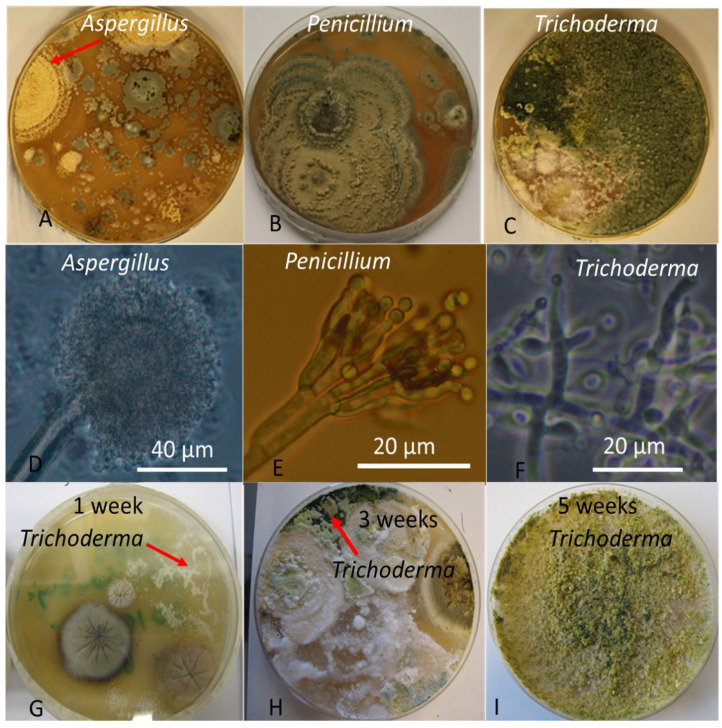
Diversity of the major toxigenic fungal genera obtained on fallout plates from the 15 schools. Panels (**A**) (arrow), (**B**,**C**) in the upper row show the dominant colony types on malt extract agar incubated for 4–6 weeks at 21 °C. The middle row: Panels (**D**–**F**) show the conidia of the colonies pictured using a phase contrast microscope. The lower row shows the mycoparasitic growth characteristic for representatives of the genus *Trichoderma*. Panel (**G**) shows lately germinated young hyphae (arrow) after 1 week of incubation. Panel (**H**) shows hyphae covering cogrowing fungi producing new green conidia (arrow). Panel (**I**) shows a 5-week-old plate completely covered by green conidia of *Trichoderma*. Representatives of mycoparasitic members of the genus *Trichoderma* covered 19% of the plates collected, and represented the dominant characteristic fungal genus.

Dust toxicity was divided into three categories and microbial toxicity into two categories [50,51]. The number of teachers with primary workspace dust toxicity results was 169; 111 (65.7%) were non-toxic (EC_50_ ≥ 25 µg/mL), 43 (25.4%) were low toxic (EC_50_ = 12 µg/mL) and 15 (8.9%) were highly toxic (EC_50_ ≤ 6 µg/mL). The number of teachers with primary workspace microbial toxicity results was 200; 118 (59%) were non-toxic (EC_50_ > 12 µg/mL), and 82 (41%) were toxic (EC_50_ ≤ 12 µg/mL). The fallout plates representative for sampling are pictured in Figure 1. The mold genera recognized based on colony morphology on MEA plates were *Penicillium, Aspergillus*, and *Trichoderma.* The dominant genera, representing *Trichoderma* isolates, were detected on 19% of the plates. The isolates were covering and feeding on co-growing fungal colonies, and apparently represent mycoparasitic species. The mycoparasitic and mycotrophic *Trichoderma* isolates covered between 5% and >50% of all plates collected in the different schools. *Trichoderma* isolates grew in 13 (15.9%) toxic fallout plates and two (1.7%) non-toxic plates (*p* < 0.001, Fisher’s exact test).

According to the logistic regression model based on dust samples and adjusted for age, gender, smoking, and atopy (Table 2), 12 work-related symptoms were statistically significantly more common among teachers whose primary classroom was highly toxic compared to non-toxic classrooms (adjusted ORs in parentheses): nose stuffiness (4.1), runny nose (6.9), hoarseness (6.4), globus sensation (9.0), throat mucus (7.6), throat itching (4.4), shortness of breath (12.2), dry cough (4.7), wet eyes (12.7), hypersensitivity to sound (7.9), difficulty falling asleep (7.6), and increased need for sleep (7.7). Based on analyses of cultured microbial fallout samples (Table 3) among teachers whose classrooms were toxic, nine work-related symptoms were statistically significantly more common compared to non-toxic classes (adjusted ORs in parentheses): headache (2.3), nose stuffiness (2.2), nose dryness (2.2), mouth dryness (2.8), hoarseness (2.2), sore throat (2.8), throat mucus (2.3), eye discharge (10.2), and increased need for sleep (3.5). ORs could not be calculated for three symptoms (nose stinging, wheezing, and exanthema) because these symptoms were not reported by teachers in the group of non-toxic microbes. Work-related nose stinging occurred in 4/82 and 0/118 teachers with and without toxic airborne microbes in their classrooms, respectively. The crude OR results are presented in the Supplementary Tables S1 and S2.

Allergic rhinitis was significantly more common among teachers in toxic classrooms with regard to dust sample results (Table 2).

## 3. Discussion

Our results show variations in intrinsic toxicities in settled dusts and fungal biomass from fallout plates collected from 231 classrooms in 15 schools in the city of Helsinki, Finland. 41% of the cultured microbial fallout samples and 34% of the wiped dust samples in classrooms were toxic in vitro. Our results suggest that exposure to toxic dust and microbes increases the risk of teachers’ work-related symptoms.

In our series, significant toxicity-related symptoms were typically linked with the respiratory tract (nose stuffiness, nose dryness, nose stinging, runny nose, allergic rhinitis, mouth dryness, hoarseness, sore throat, globus sensation, throat mucus, throat itching, shortness of breath, and dry cough) and eyes (wet eyes and eye discharge). Our results show that some of the work-related symptoms were significantly associated with both dust and microbial toxicities, whereas some toxicity-associated symptoms were identified only by dust or microbial analyses, suggesting differences in exposure or activation of body responses.

Toxic indoor exposure is a complex phenomenon [16,27,52,53,54,55]. Microbial toxins are known to be present in non-industrial buildings, but concentrations of individual toxins are typically low [21,22,23]. However, in addition to hundreds of known microbial toxins, new ones are still identified. Furthermore, a huge number of different toxic chemicals have been found in indoor dust, such as plasticizers, flame retardants, polycyclic aromatic hydrocarbons, pesticides, and other biocides [56]. The toxic properties of many chemicals are not yet known [57]. Chemical assays cannot determine the harmfulness of total exposure to microbial and anthropogenic toxins or their interactions. Thus, bioassays providing an integrated picture of overall toxicity are essential tools for understanding toxic mechanisms, detecting known and unknown toxins, and studying the potential health relevance of complex toxic exposure [37,57,58,59,60]. However, sensitivity and specificity varies in different bioassays, and there is no single comprehensive method for detecting all potentially adverse effects of indoor air on human health.

BSMI assay has been identified as a sensitive biosensor of microbial toxins in buildings associated with health complaints and food poisonings [12,16,43,46,53]. Toxicity detected in fallout plates may indicate dominance of bacteria producing sperm-toxic substances—such as the mitochondriotoxins produced by *Streptomycetes, Bacillus,* and *Paenibacillus,* [9,16,38,43,45,53,58]—and also toxins affecting ion homeostasis and energy metabolism produced by the fungal genera *Chaetomium*, *Stachybotrys,* mycoparasitic *Trichoderma* species and toxigenic *Paecilomyces, Aspergillus* and *Penicillium* species [10,11,12,46]. Many of these species and genera are recognized as indicators of water damage to buildings [61,62]. The mycoparasitic genus *Trichoderma* feed on fungi colonizing indoor spaces, and may indicate mold growth in building structures [10,41,63]. It was interesting to note that in toxic fallout plates there were significantly more mycoparasitic and mycotrophic *Trichoderma* isolates than in non-toxic plates.

Mitochondrial toxins affecting cellular energy metabolism and ion homeostasis cause sublethal injury, which can be demonstrated by a decreased motility in boar sperm [53,64,65,66]. In addition to a wide variety of different microbial toxins, also environmental pollutants, biocides, consumer chemicals, tobacco smoke, and particulate matter can damage mitochondria [47,67,68,69,70,71,72]. We have earlier seen that there was no correlation between dust and microbial toxicities [51], and we suggested that dust toxicity was at least partially derived from the environmental chemicals, whereas microbial culture toxicity directly expresses microbial toxicity.

Mitochondria play an important role in human physiological regulatory mechanisms [73,74]. In addition to energy production and cellular calcium regulation, mitochondria have a central role in the regulation of the inflammatory response through production of reactive oxygen species (mtROS) and activation of the NLRP3 inflammasome [75,76]. Mitochondrial dysfunction and ROS-mediated oxidative stress are associated with the pathophysiology of many chronic inflammatory diseases, such as asthma, and chronic obstructive pulmonary disease, as well as Alzheimer’s and Parkinson’s diseases [77,78,79]. Moreover, environmental mitochondriotoxic exposure has been suggested to be a significant causal factor for these diseases [68,69,71,76,80,81,82,83,84].

Microbial toxins cause immunological inflammation, especially in combination with other exposures, such as to lipopolysaccharides (LPS) [9,33,40,85]. Pro-inflammatory cytokines have been found to interfere with mitochondrial function [86]. Transient receptor potential (TRP) channels in the chemosensory trigeminal C-fibers innervating the nasal cavity, throat, and conjunctiva and in the vagal C-fibers innervating the lower respiratory tract can sense irritating and toxic chemicals [87,88]. The sperm-toxic microbial toxin antimycin A has been shown to cause mitochondrial dysfunction, mtROS production, and C-fiber activation via TRP channels in experimental mice models [89,90,91]. When exposed to mtROS—even non-toxic concentrations of chemicals, LPS, or inflammatory mediators—this chemosensory system can induce neurogenic inflammation via neuropeptide secretion and mast cell degranulation [90,92,93,94,95,96,97,98].

The majority of the 18 boar sperm toxicity-related symptoms may be due to one or more of these three mechanisms (1) mitochondrial dysfunction / oxidative stress, (2) immunological inflammation, and (3) chemosensory C-fibers. Five symptoms (headache, throat mucus and itching, shortness of breath, and cough) may be activated via all three mechanisms [99,100,101,102,103,104,105,106,107,108,109,110,111], six symptoms (stuffy and runny nose, hoarseness, sore throat, globus sensation, watery eyes) via immunological inflammation and C-fibers [99,106,112,113,114,115,116,117,118,119], and one symptom (difficulty falling asleep) via mitochondrial dysfunction and immunological inflammation [120,121,122]. Eye discharge and increased need for sleep can be triggered due to immunological inflammation [119,120,121], and nasal stinging via C-fibers [88]. Only three symptoms associated with boar sperm toxicity (dry nose and mouth, sound hypersensitivity) did not appear to be related to these three mechanisms according to thus far published literature. This strong overlap supports the hypothesis that mitochondrial dysfunction, immunological inflammation, and chemosensory C-fibers are involved in the pathophysiology of boar sperm toxicity-related symptoms.

Respiratory protection from toxic particles is significantly based on airway mucociliary clearance [123]. However, microbial toxins can inhibit the motility of cilia, just as occurs with exposure to tobacco smoke [59,124,125]. The airway particle load is directed mostly to the upper respiratory tract [126]. Upper respiratory symptoms were also most common among teachers’ toxicity-related symptoms in our series.

Axonal transport of the olfactory and trigeminal nerves and bulk flow through the perivascular space provide direct access to toxic exposures from the nasal cavity to the brain, which bypasses the blood–brain barrier [127]. The importance of mitochondrial toxicity and the nose-to-brain route has been demonstrated in an experimental model by nasal administration of mitochondriotoxic MPTP to induce Parkinson’s disease in mice [128]. Toxic exposures may also be transferred from the respiratory tract into systemic circulation, thus bypassing hepatic first-pass metabolism [126,129]. Thus, adverse inhalation exposure to lipophilic mitochondriotoxins may potentially cause consequences in any part of the body.

**Figure 2:**
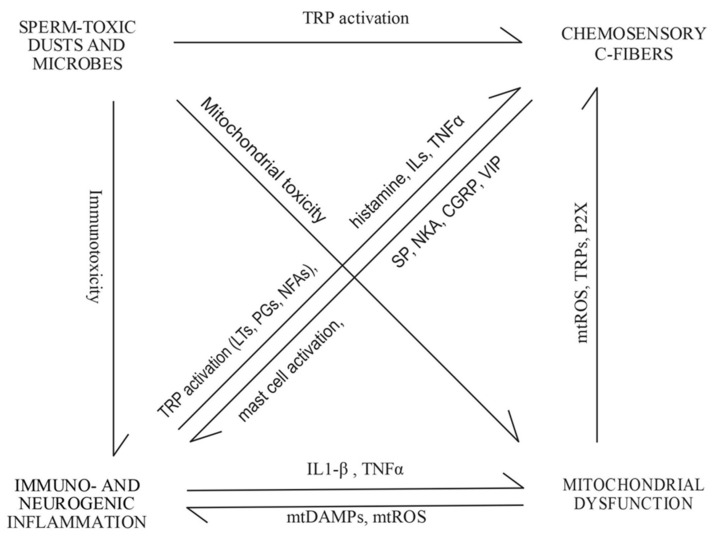
Hypothetical model of pathophysiological processes triggered by sperm-toxic dusts and microbes. The work-related symptoms are probably due to activation of described mechanisms and their interactions. Abbreviations: TRP—transient receptor potential channels; LTs—leukotrienes; PGs—prostaglandins; NFAs—nitrated fatty acids; ILs—interleukins, IL-1β—interleukin 1beta; TNFα—tumor necrosis factor alpha; SP—substance P; NKA—neurokinin A; CGRP—calcitonin gene-related peptides; VIP—vasoactive intestinal peptide; mtROS—mitochondria-derived reactive oxygen species; P2X—purinergic 2X receptor; mtDAMPs—mitochondrial damage-associated molecular patterns.

Although the pathogenesis of BRS thus far remains open, our results show that teachers have dust and microbial toxicity-associated symptoms in toxic classrooms demonstrated by BSMI assay. This assay also detects mitochondrial dysfunction, which could explain how BRS may be initiated. Several mechanisms are likely involved in the inhibition of these mechanisms, such as the anti-inflammatory effect of CGRP secreted by C-fibers [130]. Further studies are needed to explore the possible presence of mitochondrial dysfunction and oxidative stress, activation, and peripheral sensitization of chemosensory C-fibers, and neuro-immune crosstalk in those exposed to mitochondriotoxic microbes and chemicals in indoor environments. A possible link between these physiological systems and work-related symptoms is described in Figure 2.

Our study had some limitations. In this series, toxicity analyses were performed only for teachers’ current principal classrooms, while data related to exposures in other areas of school, home, and previous work places were not available. The low proportion of men (18%) weakens the assessment of the association between toxicity and symptoms in men. Furthermore, our sampling methods did not allow us to analyze the exact amount of teachers’ exposure, and BSMI assay is insensitive to many types of toxins—e.g., toxins affecting macromolecular syntheses and several mycotoxins such as sterigmatocystine and ochratoxin [46]. Quantities of toxic microbes in indoor air were not determined and systematic identification of toxic microbes was not performed. Other exposures potentially provoking symptoms—such as volatile organic compounds, room temperature, insufficient ventilation rate, or high carbon dioxide concentration—were not measured concurrently. However, although we only tested the teachers’ current principal classrooms, we found a significant association between symptoms and measured toxicity in settled dust and cultured airborne microbial biomass.

A main strength of our study was an in vitro model that has been successfully used to identify toxin producers in buildings with health complaints, and that measures a significant mechanism of environmental toxicity. Two independent sampling methods were used in the study. Due to the strong tendency of microbial toxins to aerosolize, especially in airflow [11,16,17,20], we ruled out high airflows (e.g., vacuum cleaner) for dust sampling or sample processing to ensure that toxins were not lost from samples. We also used ethanol to extract dust and microbial samples, because a large proportion of microbial toxins and indoor chemicals are lipophilic and potentially bioaccumulative [56,131]. In addition, we made a comparison based on teachers’ individual workspace toxicity instead of a building-level comparison. Examining the association between individual symptoms and toxicity allowed a preliminary assessment of a hypothetical model of the pathophysiological mechanisms involved in sperm-toxic exposure.

Teachers did not know the toxicities of their classrooms, which reduced the risk of information bias. The study population was also comparable. Teachers belonged to the same profession and the same socio-economic class, worked in the same city, and clear inclusion criteria were defined in the study. The Real Estate Department of the City of Helsinki selected the schools to represent the actual age and condition distribution of the city’s schools.

In conclusion, our results show a clear variation in in vitro toxicity in classroom environments as measured by the BSMI assay, a sensitive indicator of mitochondrial toxicity. The risk of various work-related symptoms, especially respiratory and ocular symptoms, was strongly increased among exposed teachers in classrooms with toxic dust and airborne microbes. These findings provide a new perspective for the research field of indoor adverse exposure and pathophysiological processes in exposed, symptomatic individuals. This approach can also be used in real life for detection of adverse occupational exposures at individual or organizational levels.

## 4. Materials and Methods

Materials and methods have been presented in more detail previously [50,51]. Here we present a short summary of a sub-analysis of the same dataset.

### 4.1. Schools

Fifteen schools representing construction, building services, and ventilation systems from different decades, some of which had been renovated, were selected by the Real Estate Department of the City of Helsinki. A more detailed description of schools has been presented previously [50]. The schools were built in 1924–2004 and one building was renovated in 2009. Fourteen schools had an area of 2400–8300 m^2^ and one school 474 m^2^. Concrete was the main structural material for all the schools. Thirteen schools had a mechanical exhaust air system, eight schools also had a mechanical supply air system, and one had completely natural ventilation. Several moisture damage and indoor air studies had been required in eight of the schools, one study in one school, and no concern for indoor air quality or moisture damage had been identified in six schools. This building-level information was not available to the research team during the research project.

### 4.2. Teachers

Teachers were eligible for the study if they worked at least seven hours a week in the school under study and for at least one year in the same classroom; they were not pregnant at the time of the study; and information was available about their workspace. The symptoms of students in these schools were not studied.

We sent a questionnaire to all the teachers in the 15 schools. Demographics included age, gender, smoking status, and atopy. The survey asked whether symptoms had occurred in the last 12 months. If the teacher had answered that the symptom was related to time spent in the workplace, his/her symptom was classified as work-related. In addition to all known BRS, all other symptoms with a prevalence of at least 10% and concomitant work-relatedness of at least 50% were also selected for analysis.

The privacy of the subjects was protected by conducting the survey anonymously, thus no personal identity information was collected. According to the Ethics Committee of Helsinki University Hospital, no formal approval was required for this type of anonymous study.

### 4.3. Indoor Samples

Two types of indoor samples (wiped dust and airborne microbes) were collected from each teacher’s principal school classroom [50,51]. Cotton balls were used to wipe dust samples from surfaces above the floor (e.g., above lamps and cabinets) that had last been cleaned 8–12 months earlier. Airborne microbial propagules were collected by allowing them to fall onto malt extract agar (MEA) plates for a sampling time of 1 h. Malt extract agars (malt extract 70167, Sigma-Aldrich, Merck KGaA, Darmstadt, Germany; agar 05039, Sigma-Aldrich, Merck KGaA, Darmstadt, Germany) were incubated at 22–24 °C. After 4–6 weeks of incubation, all plates were photographed and microbial biomasses were collected. Colony-forming units were not counted, but recognizable species were identified from the photographs of microbial cultures (Figure 1). To treat these two different types of samples, the wiped dust and microbial biomasses were extracted into ethanol and evaporated to dryness at 62 °C. The residues were then re-dissolved in ethanol to a concentration of 10 mg dry weight/mL [16]. Next, boar sperm were exposed to extracts of wiped dust and microbial mass for three days [50,51]. 

### 4.4. Toxicity Assay

**Figure 3:**
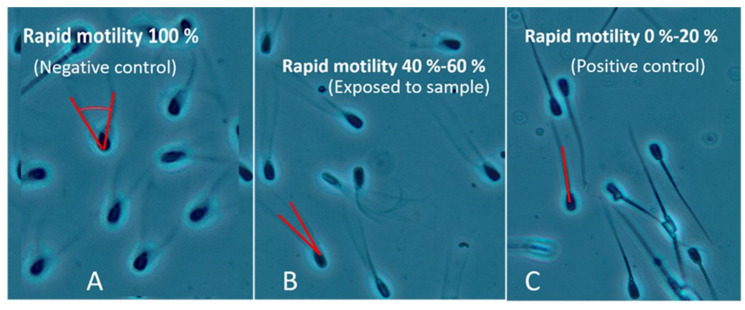
Estimation of sperm motility. Phase contrast micrographs visualizing different amplitudes of tail beating in boar sperm. The vehicle-exposed sperm cells in Panel (**A**) express high amplitude tail beating visible to the human eye as an artefact consisting of two tails. The angle between the tails is >40° and the majority of the sperm cells exhibit rapid and progressive motility. Panel (**B**) shows sperm cells with low amplitude tail beating, the angle between two tails is less than 20° and the swimming speed/the number of rapidly swimming sperm cells is reduced by 50% compared to the cells in Panel (**A**). Panel (**C**) shows the immobilized sperm cells of the positive control. In this panel, only a few sperm cells moved at all and no sperm cells with two tails were visible.

Toxicity of the extracts was tested with the classical ex vivo BSMI assay measuring sublethal toxicity as sperm motility inhibition. The BSMI assay measures inability to respond to induction of motility in resting immotile toxin-exposed sperm cells and has been described in detail earlier [12,52,53]. In brief, after exposure for three days at 22–24 °C, motility was induced in the exposed sperm cells by shaking to provide the sperm cells with oxygen and warming to 37 °C for 5 min. The induced sperm motility, i.e., progressive and rapid motility, was assessed using a phase contrast microscope with a warmed stage (37 °C) as described earlier [50]. Progressive and rapid motility was subjectively estimated as the proportion of spermatozoa exhibiting high amplitude tail beating (Figure 3A), which is required for progressive and rapid sperm motility. This is easily visualized as rapid swirling motility comparable to mass activity in a microscopic frame [53]. Static, shivering, or slowly motile sperm cells express no amplitude or low amplitude of tail beating (Figure 3B,C).

The half maximal effective concentration (EC_50_) indicated the level of toxicity (i.e., the lower the EC_50_, the higher the toxicity). The EC_50_ concentration of the ethanol-extracted dry substances in the dust- and microbial extracts is defined as the lowest concentration at which ≥50% of sperm had lost rapid and progressive motility relative to the solvent vehicle, ethanol. The motility of the ethanol-exposed sperm cells was estimated as the reference value of 100%, representing the negative control in each test. Sperm cells immobilized by exposure to triclosan exhibiting motility close to 0% were used as positive controls [37,50].

The EC_50_ (<50% motility) was estimated as the concentration between EC_< 20_ (motility > 80% compared to the control, meaning similar to the negative control) and EC_80_ (motility < 20%, similar to the immobilized control). When testing > 200 extracts, this protocol gave 99% similarity to measurements of the proportion of rapidly motile spermatozoa done with a Hamilton Thorne sperm analyzer (HTM-S, ver. 7.2; Hamilton-Thorn Research, Danvers, MA, USA). When calibrated with triclosan (Sigma Chemical Co., St. Louis, MO, USA) in 10 parallel tests, the EC_50_ was 1 μg/mL (SD ± 0.2) [37,52].

### 4.5. Statistical Analysis

The effect of toxicity on the presence of different symptoms was analyzed using a logistic regression model. The results of the logistic regression analyses are presented with odds ratios (ORs) with 95% confidence intervals (CIs). Age, sex, current smoking status, and atopy as potential confounding factors were used as adjusting variables when judging the effects of the toxicity outcomes of the classroom samples on the presence of different symptoms. In the adjustment, age was divided into four categories (≤34, 35–44, 45–54, and ≥55 years), while sex, smoking status, and atopy were dichotomous. The presence of symptoms was divided into two categories (yes or no). Dust toxicity was divided into three categories (EC_50_ ≥ 25, 12, and ≤6 µg/mL) and microbial toxicity into two categories (EC_50_ > 12 and ≤12 µg/mL). Categorical data was analyzed using Fisher’s exact test. Two-tailed *p* values are reported. All analyses were performed with SPSS for Windows (IBM Corp. Released 2019. IBM SPSS Statistics for Windows, Version 26.0. Armonk, NY: IBM Corp.).

## Figures and Tables

**Table 1:** Number of total and work-related symptoms in 231 teachers.

	Total, *N* (%)	Work-Related, *N* (%)	Literature-Known BRS, Yes or No
GENERAL SYMPTOMS			
Fatigue	88 (38.1)	65 (28.1)	yes
Headache	92 (39.8)	46 (20.0)	yes
Fever	13 (5.6)	8 (3.5)	yes
Chills	31 (13.4)	19 (8.2)	yes
Generalized feeling of sickness	60 (26.0)	46 (20.0)	no
Decreased physical condition	54 (23.4)	27 (11.7)	no
Indefinite feeling of thermoregulation failure	27 (11.7)	19 (8.2)	no
RESPIRATORY SYMPTOMS			
Nose stuffiness	106 (45.9)	64 (27.7)	yes
Nose dryness	84 (36.4)	52 (22.5)	yes
Nose stinging	22 (9.5)	7 (3.0)	yes
Bloody nasal discharge	30 (13.0)	16 (6.9)	yes
Runny nose	74 (32.0)	37 (16.0)	yes
Sneezing	66 (28.6)	39 (16.9)	yes
Mouth dryness	48 (20.8)	30 (13.0)	yes
Hoarseness	88 (38.1)	62 (26.8)	yes
Sore throat	42 (18.2)	27 (11.7)	yes
Wheezing	15 (6.5)	4 (1.7)	yes
Shortness of breath	21 (9.1)	12 (5.2)	yes
Asthma attacks	12 (5.2)	5 (2.2)	yes
Dry cough	53 (22.9)	40 (17.3)	yes
Pressure in the cheek	42 (18.2)	22 (9.5)	no
Globus sensation	31 (13.4)	19 (8.2)	no
Throat mucus	70 (30.3)	50 (21.6)	no
Throat itching	49 (21.2)	35 (15.2)	no
DERMAL SYMPTOMS			
Skin dryness	58 (25.1)	20 (8.7)	yes
Exanthema	13 (5.6)	3 (1.3)	yes
Pruritus	32 (13.9)	12 (5.2)	yes
EYE SYMPTOMS			
Eye irritation	61 (26.4)	39 (16.9)	yes
Wet eyes	30 (13.0)	15 (6.5)	yes
Dry eyes	67 (29.0)	45 (19.5)	yes
Swollen eyelids	19 (8.2)	9 (3.9)	yes
Red eyes	32 (13.9)	18 (7.8)	no
Eye discharge	24 (10.4)	14 (6.1)	no
HEARING SYMPTOMS			
Difficulty distinguishing speech in noise	51 (22.1)	27 (11.7)	no
Hypersensitivity to sound	28 (12.1)	14 (6.1)	no
SLEEPING SYMPTOMS			
Insomnia	41 (17.7)	25 (10.8)	yes
Difficulty falling asleep	42 (18.2)	21 (9.1)	yes
Increased need for sleep	34 (14.7)	18 (7.8)	no
MENTAL SYMPTOMS			
Difficulty concentrating	12 (5.2)	4 (1.7)	yes
Mental irritability	29 (12.6)	22 (9.5)	no
Decreased stress resistance	26 (11.3)	18 (7.8)	no
BUILDING-RELATED DISEASES			
Asthma	22 (9.5)	*^a^*	yes
Allergic rhinitis	32 (13.9)	*^a^*	yes

Modified from [51]. *^a^* A disease diagnosed by a physician, association with work was not studied. BRS, building-related symptoms.

**Table 2:** Results of the multivariable adjusted logistic regression models for the impact of increased toxicity levels of wiped dust in the classroom on work-related symptoms.

	EC_50_ 12 µg mL^−1^		EC_50_ ≤ 6 µg mL^−1^	
	OR (95% CI)	*p* Value	OR (95% CI)	*p* Value
GENERAL SYMPTOMS				
Fatigue	1.08 (0.46,2.53)	0.86	3.00 (0.96,9.36)	0.058
Headache	1.04 (0.41,2.65)	>0.90	1.79 (0.49,6.57)	0.38
Fever	3.75 (0.63,22.2)	0.15	2.59 (0.23,29.4)	0.44
Chills	1.50 (0.41,5.56)	0.54	1.22 (0.13,11.2)	0.86
Generalized feeling of sickness	1.11 (0.42,2.94)	0.84	3.28 (1.00,10.8)	0.050
Decreased physical condition	1.08 (0.31,3.72)	0.90	3.85 (0.99,15.0)	0.052
Indefinite feeling of thermoregulation failure	0.66 (0.13,3.30)	0.61	2.96 (0.65,13.4)	0.16
RESPIRATORY SYMPTOMS				
Nose stuffiness	1.78 (0.76,4.15)	0.18	4.08 (1.24,13.4)	0.021
Nose dryness	1.65 (0.69,3.91)	0.26	0.97 (0.24,4.01)	>0.90
Nose stinging	0.67 (0.07,6.37)	0.73	1.67 (0.17,16.8)	0.66
Bloody nasal discharge	3.33 (0.73,15.2)	0.12	2.22 (0.21,23.0)	0.51
Runny nose	4.24 (1.49,12.1)	0.0070	6.93 (1.76,27.2)	0.0056
Sneezing	1.17 (0.43,3.18)	0.76	2.07 (0.55,7.75)	0.28
Mouth dryness	1.99 (0.63,6.33)	0.24	2.29 (0.49,10.5)	0.29
Hoarseness	3.38 (1.47,7.75)	0.0041	6.42 (1.95,21.1)	0.0022
Sore throat	0.92 (0.17,4.95)	>0.90	3.80 (0.72,19.9)	0.12
Wheezing	4.43 (0.21,95.2)	0.34	7.56 (0.27,209)	0.23
Shortness of breath	3.69 (0.76,17.9)	0.10	12.2 (1.95,76.8)	0.0076
Asthma attacks	7.07 (0.55,91.3)	0.13	14.8 (1.00,219)	0.050
Dry cough	2.14 (0.81,5.70)	0.13	4.65 (1.29,16.8)	0.019
Pressure in the cheek	0.36 (0.04,3.17)	0.36	2.76 (0.46,16.6)	0.27
Globus sensation	1.34 (0.23,7.72)	0.74	9.02 (1.74,46.7)	0.0088
Throat mucus	2.46 (0.99,6.14)	0.053	7.64 (2.21,26.4)	0.0013
Throat itching	2.19 (0.71,6.75)	0.17	4.35 (1.08,17.6)	0.039
DERMAL SYMPTOMS				
Skin dryness	0.66 (0.17,2.50)	0.54	0.65 (0.07,5.55)	0.69
Exanthema	3.45 (0.15,78.0)	0.44	14.78 (0.36,606)	0.16
Pruritus	0.63 (0.06,6.29)	0.69	2.24 (0.19,26.2)	0.52
EYE SYMPTOMS				
Eye irritation	0.93 (0.30,2.90)	>0.90	3.58 (0.89,14.4)	0.072
Wet eyes	4.06 (0.65,25.3)	0.13	12.7 (1.44,112)	0.022
Dry eyes	0.86 (0.31,2.40)	0.78	1.94 (0.52,7.28)	0.32
Swollen eyelids	0.65 (0.07,6.28)	0.71	2.29 (0.21,25.5)	0.50
Red eyes	0.26 (0.03,2.46)	0.24	2.34 (0.17,31.4)	0.52
Eye discharge	0.80 (0.08,8.25)	0.85	7.69 (0.96,61.4)	0.054
HEARING SYMPTOMS				
Difficulty distinguishing speech in noise	0.14 (0.02,1.16)	0.069	2.10 (0.45,9.69)	0.34
Hypersensitivity to sound	N.D.		7.91 (1.70,36.8)	0.0084
SLEEPING SYMPTOMS				
Insomnia	1.00 (0.32,3.11)	>0.90	2.57 (0.59,11.2)	0.21
Difficulty falling asleep	1.14 (0.32,4.02)	0.84	7.58 (1.93,29.8)	0.0038
Increased need for sleep	0.22 (0.03,1.76)	0.15	7.74 (2.09,28.6)	0.0022
MENTAL SYMPTOMS				
Difficulty concentrating	N.D.		17.8 (0.64,496)	0.090
Mental irritability	0.91 (0.27,3.10)	0.88	2.53 (0.58,11.0)	0.22
Decreased stress resistance	1.42 (0.39,5.18)	0.60	3.33 (0.75,14.8)	0.11
BUILDING-RELATED DISEASES				
Asthma	2.13 (0.59,7.76)	0.25	1.44 (0.22,9.51)	0.70
Allergic rhinitis	1.07 (0.33,3.44)	0.90	4.64 (1.08,20.0)	0.039

The results are presented as odds ratios (ORs) with 95% confidence intervals (CIs). ORs are adjusted for age, gender, smoking, and atopy. *P* values are for comparisons between teachers with toxic and non-toxic classroom samples. The half maximal effective concentration (EC_50_) indicates the degree of toxicity (ie, the lower the EC_50_, the higher the toxicity). N.D., not definable.

**Table 3:** Results of the multivariable adjusted logistic regression models for the impact of toxicity of airborne microbes in the classroom on work-related symptoms.

	EC_50_ ≤ 12 µg mL^−1^	
	OR (95% CI)	*p* Value
GENERAL SYMPTOMS		
Fatigue	1.63 (0.85,3.12)	0.14
Headache	2.26 (1.06,4.79)	0.034
Fever	2.80 (0.42,18.6)	0.29
Chills	0.94 (0.31,2.85)	>0.90
Generalized feeling of sickness	1.81 (0.85,3.85)	0.12
Decreased physical condition	1.47 (0.59,3.64)	0.40
Indefinite feeling of thermoregulation failure	2.02 (0.68,5.96)	0.20
RESPIRATORY SYMPTOMS		
Nose stuffiness	2.19 (1.10,4.38)	0.026
Nose dryness	2.17 (1.05,4.52)	0.038
Nose stinging *^a^*	N.D.	
Bloody nasal discharge	2.34 (0.68,8.04)	0.18
Runny nose	2.07 (0.92,4.66)	0.79
Sneezing	1.50 (0.67,3.39)	0.33
Mouth dryness	2.76 (1.13,6.71)	0.026
Hoarseness	2.18 (1.12,4.26)	0.022
Sore throat	2.81 (1.08,7.31)	0.034
Wheezing *^a^*	N.D.	
Shortness of breath	4.79 (0.89,25.7)	0.067
Asthma attacks	1.67 (0.11,24.5)	0.71
Dry cough	1.10 (0.50,2.44)	0.81
Pressure in the cheek	2.60 (0.89,7.59)	0.079
Globus sensation	2.32 (0.76,7.05)	0.14
Throat mucus	2.28 (1.09,4.74)	0.028
Throat itching	2.22 (0.93,5.31)	0.073
DERMAL SYMPTOMS		
Skin dryness	3.02 (0.97,9.44)	0.057
Exanthema *^a^*	N.D.	
Pruritus	0.90 (0.22,3.76)	0.89
EYE SYMPTOMS		
Eye irritation	2.07 (0.94,4.56)	0.073
Wet eyes	2.97 (0.90,9.76)	0.073
Dry eyes	1.73 (0.84,3.57)	0.14
Swollen eyelids	2.23 (0.49,10.1)	0.30
Red eyes	2.70 (0.91,7.99)	0.072
Eye discharge	10.2 (2.03,50.9)	0.0048
HEARING SYMPTOMS		
Difficulty distinguishing speech in noise	1.52 (0.64,3.65)	0.34
Hypersensitivity to sound	0.72 (0.20,2.56)	0.61
SLEEPING SYMPTOMS		
Insomnia	1.85 (0.70,4.91)	0.22
Difficulty falling asleep	2.54 (0.87,7.46)	0.089
Increased need for sleep	3.54 (1.03,12.2)	0.045
MENTAL SYMPTOMS		
Difficulty concentrating	1.68 (0.19,14.6)	0.64
Mental irritability	1.10 (0.37,3.26)	0.87
Decreased stress resistance	0.98 (0.31,3.06)	>0.90
BUILDING-RELATED DISEASES		
Asthma	1.28 (0.43,3.77)	0.66
Allergic rhinitis	1.59 (0.63,4.03)	0.33

The results are presented as odds ratios (ORs) with 95% confidence intervals (CIs). ORs are adjusted for age, gender, smoking, and atopy. *P* values are for comparisons between teachers with toxic and non-toxic classroom samples. The half maximal effective concentration (EC_50_) indicates the degree of toxicity (ie, the lower the EC_50_, the higher the toxicity). N.D., not definable. *^a^* These symptoms were not reported by teachers in the group of non-toxic microbes, so OR could not be calculated.

## Data Availability

Data supporting reported results are available upon reasonable request from the correspondence author.

## Supplementary Materials

The following are available online at https://www.mdpi.com/article/10.3390/pathogens10111360/s1, Table S1: title, The results of the crude logistic regression models for the impact of increased toxicity levels of wiped dust in the classroom on work-related symptoms.; Table S2: The results of the crude logistic regression models for the impact of toxicity of airborne microbes in the classroom on work-related symptoms.

## Abbreviations

BRS	building-related symptoms
BSMI	boar sperm motility inhibition assay
CGRP	calcitonin gene-related peptide
CI	confidence interval
EC_50_	half maximal effective concentration
IL	interleukin
IL-1β	interleukin 1beta
LPS	lipopolysaccharide
LT	leukotriene
MEA	malt extract agar
MPTP	1-methyl-4-phenyl-1,2,3,6-tetrahydropyridine
mtDAMP	mitochondrial damage-associated molecular pattern
mtROS	mitochondrial-derived reactive oxygen species
NFA	nitrated fatty acid
NKA	neurokinin A
NLRP3	NLR family pyrin domain containing 3
OR	odds ratio
PG	prostaglandin
P2X	purinergic 2X receptor
SP	substance P
SD	standard deviation
TNFα	tumor necrosis factor alpha
TRP	transient receptor potential
VIP	vasoactive intestinal peptide
