# Supplementary material for: The Toxicity of Wiped Dust and Airborne Microbes in Individual Classrooms Increase the Risk of Teachers’ Work-Related Symptoms: A Cross-Sectional Study
Source: Pathogens. 2021 Oct 21;10(11):1360. doi: 10.3390/pathogens10111360 (PMC8624243; doi:10.3390/pathogens10111360)
Supplement: Supplementary file 1 [file pathogens-10-01360-s001.zip › pathogens-1368951-SI.pdf]

Supplementary material

Table S1. The results of the crude logistic regression models for the impact of increased toxicity levels of wiped dust in the classroom on work-related symptoms.

	Crude OR (95% CI)		
	$EC_{50} \geq 25$ µg mL^{-1}	EC_{50} 12 µg mL^{-1}	$EC_{50} \leq 6$ µg mL^{-1}
GENERAL SYMPTOMS			
Fatigue	1	1.02 (0.44,2.35)	2.86 (0.95,8.64)
Headache	1	1.01 (0.41,2.49)	1.56 (0.45,5.38)
Fever	1	2.77 (0.54,14.3)	2.57 (0.25,26.4)
Chills	1	1.36 (0.39,4.76)	0.92 (0.11,7.91)
Generalized feeling of sickness	1	0.97 (0.37,2.50)	3.23 (1.03,10.1)
Decreased physical condition	1	1.06 (0.31,3.59)	3.67 (0.99,13.7)
Indefinite feeling of thermoregulation failure	1	0.57 (0.12,2.74)	2.83 (0.67,11.9)
RESPIRATORY SYMPTOMS			
Nose stuffiness	1	1.53 (0.68,3.45)	3.35 (1.10,10.2)
Nose dryness	1	1.44 (0.63,3.30)	1.01 (0.26,3.90)
Nose stinging	1	0.65 (0.07,6.01)	1.91 (0.20,18.3)
Bloody nasal discharge	1	2.82 (0.67,11.8)	1.91 (0.20,18.3)
Runny nose	1	4.02 (1.46,11.1)	6.44 (1.77,23.4)
Sneezing	1	1.11 (0.42,2.89)	2.01 (0.57,7.06)
Mouth dryness	1	1.68 (0.57,4.96)	2.52 (0.61,10.5)
Hoarseness	1	2.98 (1.35,6.60)	5.53 (1.79,17.1)
Sore throat	1	0.64 (0.13,3.16)	3.22 (0.75,13.8)
Wheezing	1	2.68 (0.16,43.9)	2.68 (0.16,43.9)
Shortness of breath	1	3.79 (0.81,17.7)	9.00 (1.63,49.6)
Asthma attacks	1	5.50 (0.49,62.3)	16.9 (1.43,200)
Dry cough	1	1.89 (0.75,4.77)	4.62 (1.43,15.0)
Pressure in the cheek	1	0.31 (0.04,2.59)	1.98 (0.38,10.3)
Globus sensation	1	1.06 (0.20,5.69)	7.71 (1.80,33.0)
Throat mucus	1	2.11 (0.88,5.02)	6.79 (2.16,21.3)
Throat itching	1	1.82 (0.65,5.06)	4.55 (1.31,15.7)
DERMAL SYMPTOMS			
Skin dryness	1	0.63 (0.17,2.37)	0.59 (0.07,4.89)
Exanthema	1	2.68 (0.16,43.9)	7.86 (0.47,132)
Pruritus	1	0.43 (0.05,3.66)	1.25 (0.14,11.2)
EYE SYMPTOMS			
Eye irritation	1	1.02 (0.34,3.06)	2.74 (0.76,9.88)
Wet eyes	1	2.77 (0.54,14.3)	5.54 (0.85,36.3)
Dry eyes	1	0.81 (0.30,2.18)	1.76 (0.51,6.12)
Swollen eyelids	1	0.65 (0.07,6.01)	1.91 (0.20,18.3)
Red eyes	1	0.43 (0.05,3.66)	1.25 (0.14,11.2)
Eye discharge	1	0.88 (0.09,8.68)	5.54 (0.85,36.3)
HEARING SYMPTOMS			
Difficulty distinguishing speech in noise	1	0.17 (0.02,1.33)	1.73 (0.43,6.91)
Hypersensitivity to sound	1	N.D.	6.36 (1.55,26.0)
SLEEPING SYMPTOMS			
Insomnia	1	1.11 (0.37,3.38)	2.06 (0.51,8.36)
Difficulty falling asleep	1	1.19 (0.35,4.10)	5.67 (1.59,20.2)
Increased need for sleep	1	0.22 (0.03,1.77)	6.06 (1.81,20.2)

MENTAL SYMPTOMS			
Difficulty concentrating	1	N.D.	3.89 (0.33,45.7)
Mental irritability	1	0.87 (0.26,2.86)	2.06 (0.51,8.36)
Decreased stress resistance	1	1.36 (0.39,4.76)	3.22 (0.75,13.8)
BUILDING-RELATED DISEASES			
Asthma	1	1.53 (0.48,4.87)	1.74 (0.34,8.96)
Allergic rhinitis	1	0.86 (0.29,2.55)	2.33 (0.66,8.26)

The results are presented as odds ratios (ORs) with 95% confidence intervals (CIs).
N.D., not definable

Table S2. The results of the crude logistic regression models for the impact of toxicity of airborne microbes in the classroom on work-related symptoms.

	Crude OR (95% CI)
	EC_{50} ≤12 μg mL^{-1}
GENERAL SYMPTOMS	
Fatigue	1.61 (0.86,3.01)
Headache	2.08 (1.02,4.25)
Fever	2.23 (0.36,13.7)
Chills	1.15 (0.41,3.21)
Generalized feeling of sickness	1.95 (0.95,4.00)
Decreased physical condition	1.69 (0.71,4.05)
Indefinite feeling of thermoregulation failure	1.98 (0.71,5.56)
RESPIRATORY SYMPTOMS	
Nose stuffiness	1.96 (1.03,3.73)
Nose dryness	2.07 (1.04,4.11)
Nose stinging [a]	N.D.
Bloody nasal discharge	2.14 (0.65,6.99)
Runny nose	1.69 (0.78,3.65)
Sneezing	1.33 (0.61,2.91)
Mouth dryness	2.21 (0.96,5.10)
Hoarseness	2.07 (1.10,3.91)
Sore throat	2.75 (1.14,6.64)
Wheezing [a]	N.D.
Shortness of breath	4.64 (0.91,23.6)

Asthma attacks	2.96 (0.26,33.2)
Dry cough	1.11 (0.53,2.33)
Pressure in the cheek	2.23 (0.81,6.14)
Globus sensation	1.98 (0.71,5.56)
Throat mucus	1.94 (0.98,3.84)
Throat itching	1.84 (0.82,4.10)
DERMAL SYMPTOMS	
Skin dryness	3.55 (1.18,10.6)
Exanthema [a]	N.D.
Pruritus	0.97 (0.26,3.55)
EYE SYMPTOMS	
Eye irritation	2.10 (1.00,4.44)
Wet eyes	2.82 (0.91,8.77)
Dry eyes	1.82 (0.91,3.67)
Swollen eyelids	2.52 (0.59,10.8)
Red eyes	3.25 (1.16,9.05)
Eye discharge	8.17 (1.74,38.4)
HEARING SYMPTOMS	
Difficulty distinguishing speech in noise	1.42 (0.63,3.21)
Hypersensitivity to sound	0.71 (0.21,2.46)
SLEEPING SYMPTOMS	
Insomnia	1.90 (0.75,4.83)
Difficulty falling asleep	2.93 (1.04,8.29)
Increased need for sleep	4.01 (1.21,13.3)
MENTAL SYMPTOMS	
Difficulty concentrating	1.47 (0.20,10.6)
Mental irritability	1.15 (0.41,3.21)
Decreased stress resistance	1.10 (0.37,3.30)
BUILDING-RELATED DISEASES	

Asthma	1.35 (0.52,3.49)
Allergic rhinitis	1.54 (0.68,3.53)

The results are presented as odds ratios (ORs) with 95% confidence intervals (CIs).

N.D., not definable

[a] These symptoms were not reported by teachers in the group of non-toxic microbes, so OR could not be calculated.
